# Risk factors for early recurrence in patients with pancreatic ductal adenocarcinoma who underwent curative resection

**DOI:** 10.1186/s12957-023-03141-3

**Published:** 2023-08-24

**Authors:** Masaaki Murakawa, Shinnosuke Kawahara, Daishi Takahashi, Yuto Kamioka, Naoto Yamamoto, Satoshi Kobayashi, Makoto Ueno, Manabu Morimoto, Sho Sawazaki, Hiroshi Tamagawa, Takashi Ohshima, Norio Yukawa, Yasushi Rino, Soichiro Morinaga

**Affiliations:** 1 Department of Gastrointestinal Surgery, Kanagawa Cancer Center, Yokohama City, 241-8515 Japan; 2 Department of Gastroenterology, Kanagawa Cancer Center, Yokohama, Japan; 3 Department of Surgery, Yokohama City University School of Medicine, Yokohama, Japan

**Keywords:** Pancreatic ductal adenocarcinoma, Early recurrence, Risk factors, Neoadjuvant therapy

## Abstract

**Background:**

Pancreatic ductal adenocarcinoma (PDAC) is one of the most lethal cancers, and surgical resection is the only potentially curative approach. However, the rate of recurrence remains high, particularly within the first 6 months, and is associated with a poor prognosis. The present study evaluated the clinical characteristics and risk factors for early recurrence in pancreatic ductal adenocarcinoma (PDAC) patients who underwent curative resection, regardless of the use of neoadjuvant chemotherapy, to identify predictive factors associated with early recurrence and poor outcomes as well as to determine the optimal treatment strategy for patients at high risk of early recurrence after surgical resection.

**Methods:**

Patients who underwent pancreatic resection for PDAC at our institution from 2013 to 2021 were included in this study. We investigated the clinicopathological features of patients in groups: those with recurrence within 6 months, recurrence between 6 and 12 months, and recurrence beyond 12 months or no recurrence. A logistic regression analysis identified covariates associated with early recurrence at 6 and 12 months.

**Results:**

The study included 403 patients with a median follow-up of 25.7 months. Recurrence was observed in 279 patients, with 14.6% recurring within 6 months, 23.3% within 6–12 months, and 62% after 12 months or not at all. The preoperative CA19-9 level, modified Glasgow prognostic score (mGPS), and positive peritoneal cytology were significant risk factors for early recurrence within 6 months, while positive peritoneal cytology, lymph node metastasis, and the absence of adjuvant chemotherapy were significant risk factors for recurrence within 12 months. For patients who received preoperative chemotherapy or chemoradiotherapy, the preoperative CA19-9 level, mGPS, and positive peritoneal cytology were significant independent risk factors for early recurrence within 6 months, while positive peritoneal cytology, lymph node metastasis, and the absence of adjuvant chemotherapy were significant independent risk factors for recurrence within 12 months. The study concluded that the overall survival after surgical resection for potentially resectable PDAC worsened according to the number of risk factors present in the patient.

**Conclusions:**

We clarified that preoperative CA19-9, positive peritoneal cytology, and the lack of adjuvant chemotherapy were consistent predictors for early recurrence within 6 and 12 months. In addition, an increased number of risk factors affecting the patient was associated with a poorer overall survival after potentially curable resection. Calculating the number of risk factors for early recurrence may be an essential predictive factor when considering treatment strategies.

## Introduction

Pancreatic ductal adenocarcinoma (PDAC) is one of the most lethal cancers, representing the seventh leading cause of cancer-related death worldwide [1]. At the time of the diagnosis, 50–55% of patients have metastatic disease, 30–35% have locally advanced unresectable disease, and only the remaining 15 to 20% of patients are resectable (R) or borderline resectable (BR) [2, 3]. Surgical resection is the only potentially curative approach, but 5-year survival rates after resection for PDAC remain around 20% [3].

However, the outcomes of resected PDAC patients have gradually improved, thanks to recent advances in perioperative chemotherapy. Several clinical trials have demonstrated the clinical benefits of adjuvant chemotherapy in patients with PDAC [4–8], and current guidelines strongly recommend the use of adjuvant chemotherapy for patients with PDAC [9, 10]. There is now a broad consensus supporting the use of neoadjuvant chemotherapy (NAC) for patients with BR- and R-PDAC with high-risk features [11, 12].

Several retrospective studies have suggested potential benefits of NAC over upfront surgery, and patients with high-risk features but still R-PDAC may be considered candidates for NAC. The recent phase III PREOPANC trial demonstrated an overall survival (OS) benefit of neoadjuvant gemcitabine-based chemoradiotherapy over upfront surgery in patients with R- and BR-PDAC [13]. In addition, the initial report of the Japan Prep-02/JSAP-05 trial, a multicenter randomized phase II/III trial, showed that patients with R-PDAC who received neoadjuvant chemotherapy with gemcitabine and S-1 had a superior median OS to patients who underwent upfront surgery [14]. These results support the efficacy of NAC in patients with R-PDAC.

However, despite recent advances in perioperative adjuvant therapy for PDAC patients, the rate of recurrence after surgical resection remains high, and a certain number of patients experience early postoperative recurrence. Previous clinical trials and large cohort retrospective studies have reported recurrence rates of 66 to 80% after pancreatic cancer surgery, with 25 to 38% of recurrences occurring within 6 months of the surgery [15–17]. In particular, early postoperative recurrence is associated with a poor prognosis in patients. Early recurrence after resection was significantly associated with a worse OS than non-early recurrence after resection [16]. Furthermore, early recurrence was also shown to be associated with the post-recurrence survival [18]. Although the exact reasons for early recurrence in PDAC are not fully understood, it may be caused by the presence of potential distant metastasis at the time of resection and/or the biological characteristics of PDAC that result in more rapid progression [12, 17].

Several studies have investigated risk factors to predict early recurrence, which is crucial in PDAC patients. A previous study showed that the Charlson Age Comorbidity Index (CACI), tumor size, CA19-9 level, and modified Glasgow prognostic score (mGPS) were predictors of early recurrence in PDAC patients who underwent upfront resection [16, 18]. A Dutch multicenter study included patients who underwent PDAC resection with or without NAC, and although only 9% had received NAC, the CA19-9 level was associated with recurrence within 3 months, 3–6 months, and 6–12 months [17]. Furthermore, in patients who received preoperative chemoradiotherapy, the preoperative CA19-9 level was the only predictor of early recurrence within 12 months postoperatively [17]. Although efforts have been made to define early recurrence and identify risk factors, the optimal threshold and factors to differentiate early from late recurrence are still under discussion [16, 18], and predictors for early recurrence have yet to be clarified.

As growing evidence supports administering NAC to patients with potentially resectable PDAC, more PDAC patients are expected to receive NAC. Nevertheless, the risk factors for early recurrence among patients undergoing NAC have not yet been thoroughly investigated [16–19].

We therefore evaluated the clinical characteristics and risk factors for recurrence within 6 months, 6–12 months, and beyond 12 months in resected PDAC patients, including those receiving NAC.

## Methods

### Data collection

Four-hundred and sixty consecutive patients who underwent pancreatic resection for PDAC at our institution from January 2013 to December 2021 were enrolled in this study. The surgical procedures performed were subtotal stomach-preserving pancreatoduodenectomy (SSPPD), distal pancreatectomy combined with splenectomy (DP), and total pancreatectomy (TP) with lymph node dissection. Exclusion criteria for this study included patients with pathologically diagnosed stage IV disease, those with macroscopically positive margin resection, and patients who underwent conversion surgery for initially unresectable (UR) PDAC. Patients who underwent resection were pathologically diagnosed with stage IV disease, and those who underwent macroscopically margin-positive resection and underwent conversion surgery for initially unresectable (UR) PDAC were excluded. Perioperative demographic data were extracted from prospectively maintained institutional databases, including the age, sex, body mass index (BMI), delivery of NAC, preoperative tumor marker values, and resectability (defined according to the NCCN guidelines) [10]. The mGPS was calculated as described in a previous report [20]. Clinically significant postoperative complications were defined as those of grade ≥ IIIa according to the Clavien-Dindo classification [21].

### Survival time and follow-up

The overall survival (OS) was defined as the period from pancreatic resection to the date of death or last follow-up. The diagnosis of PDAC recurrence was confirmed pathologically, diagnosed radiologically, or comprehensively determined using multiple modalities, such as positron emission tomography (PET). The recurrence-free survival (RFS) was defined as the duration from resection to the date of the diagnosis of recurrence or the date of last follow-up if there had been no recurrence. Patients underwent computed tomography and blood sampling, including tumor marker evaluations, every 3 months for the first 3 years after surgery and every 6 months until 4 to 5 years after surgery.

### Definitions of early recurrence

As the optimal threshold for differentiating between early and late recurrence after surgery is still controversial [16, 18], we investigated the risk factors for early recurrence within either 6 or 12 months as well as the clinicopathological features in 3 groups: those with recurrence within 6 months, between 6 and 12 months, and beyond 12 months.

### Statistical analyses

All statistical analyses were performed with EZR (Saitama Medical Center, Jichi Medical University, Saitama, Japan), a graphical user interface for R (the R Foundation for Statistical Computing, Vienna, Austria) [22]. The chi-square test or Fisher’s exact test was used to evaluate the categorical variables. When appropriate, the continuous variables were analyzed using Kruskal–Wallis test or the Mann–Whitney test. For univariate and multivariate analyses, a logistic regression analysis was conducted to identify covariates associated with the incidence of early recurrence within 6 and 12 months. A receiver operating characteristic (ROC) curve and associated area under the curve (AUC) based on the OS were used to assess the cut-off values for the serum CA19-9 level and tumor size.

## Results

### Patient characteristics and classification

**Fig. 1:**
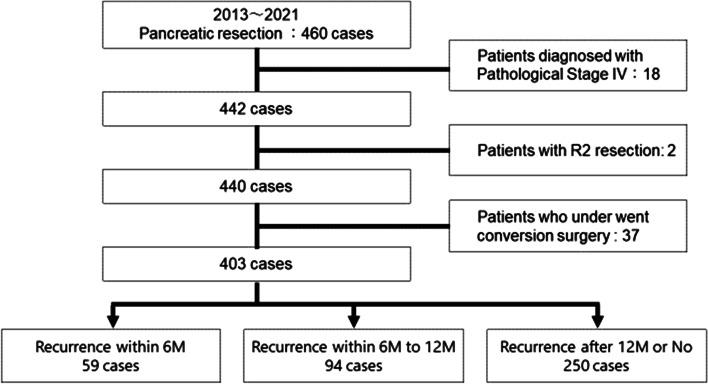
Study diagram of patient inclusion in the current study

A total of 403 patients who underwent pancreatic resection for PDAC at our institution from 2013 to 2021 were included after excluding 18 patients diagnosed with pathological stage IV disease, 2 with R2 resection, and 37 with conversion surgery (Fig. 1). The median follow-up time was 25.7 (interquartile range (IQR) 15.1–41.5) months. Recurrence was observed in 279 patients (69.2%) at the time of the analysis, and the median (IQR) time to recurrence was 14.9 (13.4–16.1) months. The patients were classified based on the time to recurrence. Fifty-nine patients (14.6%) had recurrence within 6 months, 94 (23.3%) experienced recurrence within 6 to 12 months, and 250 (62.0%) experienced recurrence after 12 months or had no recurrence.

### Patient characteristics according to the RFS

Patient characteristics are shown in Table 1. In a comparison of the groups, there were no marked differences in the administration or regimen of NAC, type of surgical procedure, or postoperative complications, including pancreatic fistula. However, the early recurrence group tended to include more BR-PDAC patients, have a higher mGPS, and have a greater proportion receiving vascular resection, a larger tumor size, poorer histology, and more advanced pathological TNM classification than the non-early recurrence group. In addition, the induction rate of adjuvant chemotherapy was only 62.7% in the early recurrence group within 6 months. In contrast, 96% of late or no recurrence patients received adjuvant chemotherapy. Reasons for the absence of adjuvant chemotherapy included early recurrence that required changing the therapeutic strategy, a poor performance status after surgery, prolonged complications (e.g., cholangitis), and patient refusal.

**Table 1:** Demographic and clinicopathological features of 403 patients who underwent PDAC resection categorized according to recurrence-free time

		Recurrence-free duration			
		<6 months (n=59)	6 to 12 months (n=94)	no rec or later (n=250)	*p-v*alue
Age in years, median(IQR)		71 (64-75)	70 (62.25-75.75)	71 (66-76.75)	0.435
Gender, n**(%)**	Male	34 (57.6%)	60 (63.8%)	116 (46.4%)	*0.01*
	Female	25 (42.4%)	34 (36.2%)	134 (53.6%)	
BMI		21.73	21.64	21.50	0.844
Neoadjuvant therapy, n**(%)**	Yes	22 (37.3%)	37 (39.4%)	84 (33.6%)	0.58
	No	37 (62.7%))	57 (60.6%)	166 (66.4%)	
NAC regimen, n**(%)**	GS	13 (56.5%)	26 (70.2%)	58 (68.2%)	0.322
	GnP	7 (30.4%)	8 (21.6%)	13 (15.3%)	
	GEM	0 (0%)	1 (2.7%)	2 (2.4%)	
	S-1+Radiation	2 (8.7%)	2 (5.4%)	11 (12.9%)	
preoperative CA19-9(U/l), median(IQR)		331.5 (84.75 – 1354.25)	165.6 (51.25 – 877.65)	70.9 (16.2 – 389.85)	*<0.001*
mGPS, n**(%)**	0	38 (64.4%)	67 (71.3%)	204 (81.6%)	*0.001*
	1	10 (16.9%)	21 (22.3%)	33 (13.2%)	
	2	11 (18.6%)	6 (6.4%)	13 (5.2%)	
Resectability, n**(%)**	R	44 (74.6%)	66 (70.2%)	208 (83.2%)	*0.021*
	BR	15 (25.4%)	28 (29.8%)	42 (16.8%)	
Surgical procedure, n**(%)**	SSPPD	34 (57.6%)	64 (68.1%)	169 (67.6%)	0.643
	DP	21 (35.6%)	25 (26.6%)	73 (29.2%)	
	TP	4 (6.8%)	5 (5.3%)	8 (3.2%)	
Vascular resection, n**(%)**	No	43 (72.9%)	58 (61.7%)	196 (78.4%)	*0.004*
	Venous	14 (23.7%)	36 (38.3%)	52 (20.8%)	
	Arterial	2 (3.4%)	0 (0%)	2 (0.8%)	
Major complication (Clavien-Dindo>IIIa), n**(%)**	Yes	21 (35.6%)	22 (23.4%)	53 (21.2%)	0.065
	No	38 (64.4%)	72 (76.6%)	197 (78.8%)	
POPF, n**(%)**	0	41 (69.5%)	58 (61.7%)	154 (61.6%)	0.106
	BL	2 (3.4%)	15 (16%)	31 (12.4%)	
	B	14 (23.7%)	19 (20.2%)	64 (25.6%)	
	C	2 (3.4%)	2 (2.1%)	1 (0.4%)	
Size of tumor (mm), median(IQR)		38 (31.5 – 51)	32 (28 – 45)	30 (23 – 38)	*<0.001*
Histological type, n**(%)**	tub	34 (57.6%)	76 (80.8%)	205 (82.0%)	*<0.001*
	por	17 (28.8%)	15 (16.0%)	28 (11.2%)	
	others	8 (13.6%)	3 (3.2%)	17 (6.8%)	
T factor TNM 8th edition, n**(%)**	1	3 (5.1%)	9 (9.6%)	47 (18.8%)	*<0.001*
	2	29 (49.2%)	60 (63.8%)	154 (61.6%)	
	3	27 (45.8%)	25 (26.6%)	49 (19.6%)	
N factor TNM 8th edition, n**(%)**	0	12 (20.3%)	19 (20.2%)	101 (40.4%)	*<0.001*
	1	24 (40.7%)	43 (45.7%)	108 (43.2%)	
	2	23 (39.0%)	32 (34.0%)	41 (16.4%)	
Peritoneal cytology, n**(%)**	positive	13 (22.4%)	10 (10.8%)	10 (4.0%)	*<0.001*
	negative	45 (77.6%)	83 (89.2%)	238 (96.0%)	
Resection margin, n**(%)**	positive	17 (28.8%)	25 (26.6%)	48 (19.2%)	0.147
	negative	42 (71.2%)	69 (73.4%)	202 (80.8%)	
Duration of hospital stay, median(IQR)		16 (13 – 23)	17 (13.25 – 23)	17 (13.5 – 28.5)	0.487
Adjuvant chemotherapy, n**(%)**	Yes	37 (62.7%)	88 (93.6%)	240 (96.0%)	*0.001*
	No	22 (37.3%)	6 (6.4%)	10 (4.0%)	
Recurrence cite, n**(%)**	Liver	27 (45.8%)	26 (27.7%)	18 (14.2%)	*<0.001*
	Multiple	14 (23.7%)	26 (27.7%)	24 (18.9%)	
	Local	5 (8.5%)	17 (18.1%)	27 (21.3%)	
	Lung	3 (5.1%)	5 (5.3%)	25 (19.7%)	
	Peritoneum	7 (11.9%)	6 (6.4%)	11 (8.7%)	
	Lymph node	3 (5.1%)	13 (13.8%)	21 (16.5%)	
	Others	0 (0%)	1 (1.1%)	1 (0.8%)	
Treatment after recurrence, n**(%)**	Yes	47 (79.7%)	82 (87.2%)	114 (90.5%)	0.123
	No	12 (20.3%)	12 (12.8%)	12 (9.5%)	

*IQR *Interquartile range, *BMI* Body mass index, *R* Resectable, *BR* Borderline resectable, *mGPS* Modified Glasgow prognostic score, *SSPPD* Subtotal stomach-preserving pancreaticoduodenectomy, *DP* Distal pancreatectomy, *TP* Total pancreatectomy, *POPF* Postoperative pancreatic fistula

Regarding the type of recurrence, the frequency of liver metastasis was higher in the group that recurred within 6 months (45.8%) than in the other groups. The frequency of local and lymph node metastasis was higher in both groups that recurred within 6 to 12 months and recurred beyond 12 months or no rec.

However, the frequency of multiple organ metastases was similar among the 3 groups (< 6 months: 23.7%, 6 to 12 months: 27.7%, and beyond 12 months or no rec.: 18.9%, respectively).

### The OS after surgery according to the recurrence timing

**Fig. 2:**
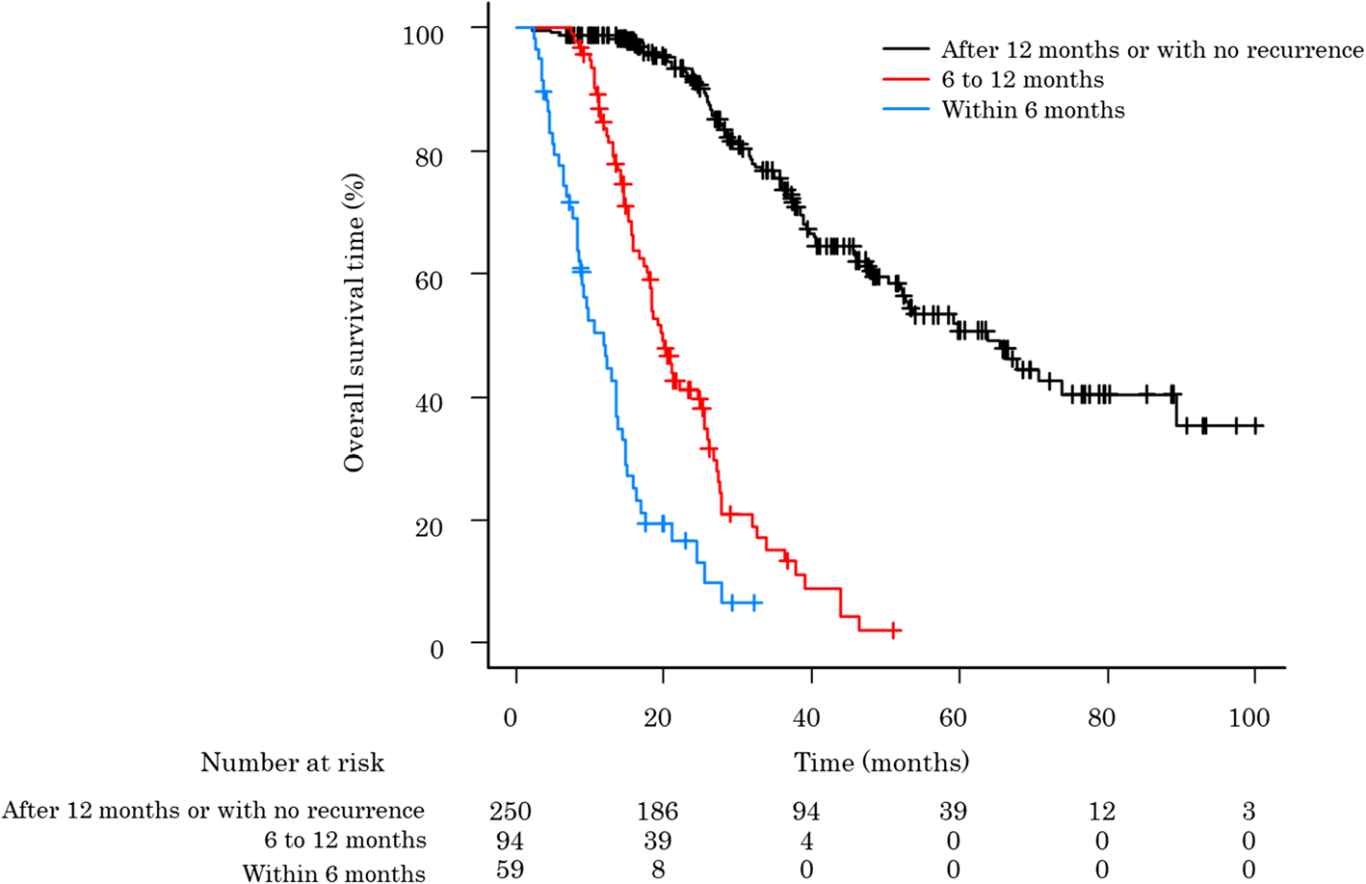
Comparison of the survival rate after surgery according to the timing of recurrence

Earlier recurrence was associated with a poorer OS after surgery (Fig. 2). Patients’ median survival time (MST) for those with recurrence within 6 months, 6 to 12 months, and after 12 months or with no recurrence was 11.6, 19.8, and 63.8 months, respectively.

### An ROC analysis to determine cut-off values for predicting early recurrence

The cut-off values of the preoperative CA19-9 level and tumor size were determined using an ROC analysis based on the OS. Among all patients, 26 (6.4%) were deemed Lewis antigen negative and excluded from the analysis. The cut-off value of the preoperative CA19-9 level was 175.8 U/ml (*AUC*: 0.642, sensitivity, 62.1%, specificity, 65.5%), and the cut-off value of the tumor size was 31 mm (*AUC*: 0.681, sensitivity 54.9%, specificity 76.3%).

### Perioperative risk factors for early recurrence in all patients

Risk factors for early recurrence within either 6 or 12 months after surgery were analyzed in all patients. On a univariate analysis, the preoperative CA19-9 level, mGPS, tumor size, postoperative complications, positive peritoneal cytology, lymph node metastasis, and the absence of adjuvant chemotherapy were associated with recurrence within 6 months. A multivariate analysis then revealed that the preoperative CA19-9 level, tumor size, postoperative complications, positive peritoneal cytology, and the absence of adjuvant chemotherapy were significant independent risk factors for recurrence within 6 months (Table 2). Regarding recurrence within 12 months, a univariate analysis showed that the preoperative CA19-9 level, mGPS, tumor size, histology, positive peritoneal cytology, lymph node metastasis, and the absence of adjuvant chemotherapy were associated with recurrence. The multivariate analysis then revealed the preoperative CA19-9 level, histology, positive peritoneal cytology, lymph node metastasis, and the absence of adjuvant chemotherapy to be significant independent risk factors (Table 3).

**Table 2:** Univariate and multivariate analyses of risk factors for recurrence within 6 months after pancreatic resection

		Univariate analysis			Multivariate analysis		
		*OR*	*95% CI*	*p-v*alue	*OR*	*95% CI*	*p-v*alue
Neoadjuvant treatment	Yes	1.065	0.629 – 1.806	0.814			
	No						
Preoperative CA19-9	< 175.8	2.005	1.441 – 2.791	*<0.001*	2.315	1.288 – 4.162	*0.005*
	≥ 175.8						
mGPS	0	1.785	1.272 – 2.505	*<0.001*	1.288	0.873 – 1.899	0.202
	1,2						
Tumor size	< 31	3.371	1.876 – 6.058	*<0.001*	1.93	1.002 – 3.719	*0.049*
	≥ 31						
Complication	Yes	1.905	1.118 – 3.246	*0.017*	2.011	1.13 – 3.577	*0.018*
	No						
Histology	tub	1.43	0.809 – 2.53	0.219			
	por, others						
Resection margin	Positive	1.461	0.831 – 2.566	0.188			
	Negative						
Peritoneal cytology	Positive	3.881	2.092 – 7.199	*<0.001*	2.31	1.142 – 4.67	*0.02*
	Negative						
N factor	0	1.726	1.217 – 2.448	*0.002*	1.19	0.8 – 1.771	0.389
	1,2						
Adjuvant chemotherapy	Yes	9.263	5.451 – 15.74	*<0.001*	7.239	3.918 – 13.38	*<0.001*
	No						

*OR* Odds ratio, *CI* Confidence interval, *mGPS* Modified Glasgow prognostic factor

**Table 3:** Univariate and multivariate analyses of risk factors for recurrence within 12 months after pancreatic resection

		Univariate analysis			Multivariate analysis		
		*OR*	*95% CI*	*p-v*alue	*OR*	*95% CI*	*p-*value
Neoadjuvant treatment	Yes	1.224	0.884 – 1.696	0.223			
	No						
Preoperative CA19-9	< 175.8	2.005	1.441 – 2.791	*<0.001*	1.658	1.173 – 2.344	*0.004*
	≥ 175.8						
mGPS	0	1.512	1.199 – 1.906	*0.001*	1.259	0.973 – 1.629	0.079
	1,2						
Tumor size	< 31	1.897	1.372 – 2.623	*<0.001*	1.36	0.948 – 1.951	0.095
	≥ 31						
Histology	tub	1.563	1.086 – 2.249	*0.01*	1.595	1.108 – 2.296	*0.001*
	por, others						
Complication	Yes	1.356	0.954 – 1.93	0.09			
	No						
Resection margin	Positive	1.416	0.993 – 2.02	0.055			
	Negative						
Peritoneal cytology	Positive	3.881	2.092 – 7.199	*<0.001*	2.579	1.595 – 4.172	*<0.001*
	Negative						
N factor	0	1.76	1.418 – 2.183	*<0.001*	1.785	1.148 – 2.775	*0.01*
	1,2						
Adjuvant chemotherapy	No	4.912	3.247 – 7.432	*<0.001*	4.799	3.036 – 7.586	*<0.001*
	Yes						

*OR* Odds ratio, *CI* Confidence interval, *mGPS* Modified Glasgow prognostic factor

### Perioperative risk factors for early recurrence in patients who received preoperative therapy

The risk factors for early recurrence within 6 or 12 months after surgery were investigated in patients who received preoperative chemotherapy or chemoradiotherapy. Multivariate analyses following univariate analyses detected preoperative CA19-9, mGPS, and positive peritoneal cytology as significant independent risk factors for recurrence within 6 months (Table 4) and positive peritoneal cytology, lymph node metastasis, and the absence of adjuvant chemotherapy for recurrence within 12 months (Table 5).

**Table 4:** Univariate and multivariate analyses of risk factors for recurrence within 6 months after pancreatic resection with preoperative therapy

		Univariate analysis			Multivariate analysis		
		*OR*	*95% CI*	*p-v*alue	*OR*	*95% CI*	*p-v*alue
Preoperative CA19-9	< 175.8	1.637	0.93 – 2.88	0.087			
	≥ 175.8						
mGPS	0	1.475	0.98 – 2.22	0.061			
	1,2						
Tumor size	< 31	1.845	1.11 – 3.08	*0.01*	1.482	0.85 – 2.58	0.402
	≥ 31						
Complication	Yes	1.218	0.686 – 2.16	0.501			
	No						
Histology	tub	1.395	0.765 – 2.55	0.278			
	por, others						
Resection margin	Positive	1.365	0.737 – 2.53	0.322			
	Negative						
Peritoneal cytology	Positive	4.921	2.3 – 10.5	*<0.001*	2.77	1.17 – 6.55	*0.02*
	Negative						
N factor	0	1.805	1.28 – 2.55	*<0.001*	1.52	1.04 – 2.23	*0.03*
	1,2						
Adjuvant chemotherapy	No	3.266	1.4 – 7.62	*0.006*	2.509	1.00 – 6.29	*0.049*
	Yes						

*OR* Odds ratio, *CI* Confidence interval, *mGPS* Modified Glasgow prognostic factor

**Table 5:** Univariate and multivariate analyses of risk factors for recurrence within 12 months after pancreatic resection with preoperative therapy

		Univariate analysis			Multivariate analysis		
		*OR*	*95% CI*	*p-v*alue	*OR*	*95% CI*	*p-v*alue
Preoperative CA19-9	< 175.8	1.637	0.93–2.881	0.087			
	≥ 175.8						
mGPS	0	1.475	0.982–2.216	0.061			
	1.2						
Tumor size	< 31	1.845	1.107–3.077	*0.01*	1.482	0.853–2.575	0.402
	≥ 31						
Complication	Yes	1.218	0.686–2.162	0.501			
	No						
Resection margin	Positive	1.365	0.737–2.527	0.322			
	Negative						
Peritoneal cytology	Positive	4.921	2.3–10.53	< *0.001*	2.77	1.172–6.548	*0.02*
	Negative						
N factor	0	1.805	1.278–2.548	< *0.001*	1.52	1.036–2.231	*0.03*
	1.2						
Adjuvant chemotherapy	No	3.266	1.399–7.624	*0.006*	2.509	1.00–6.292	*0.049*
	Yes						

*OR* Odds ratio, *CI* Confidence interval, *mGPS* Modified Glasgow prognostic factor

### Number of risk factors and the OS after pancreatic resection

**Fig. 3:**
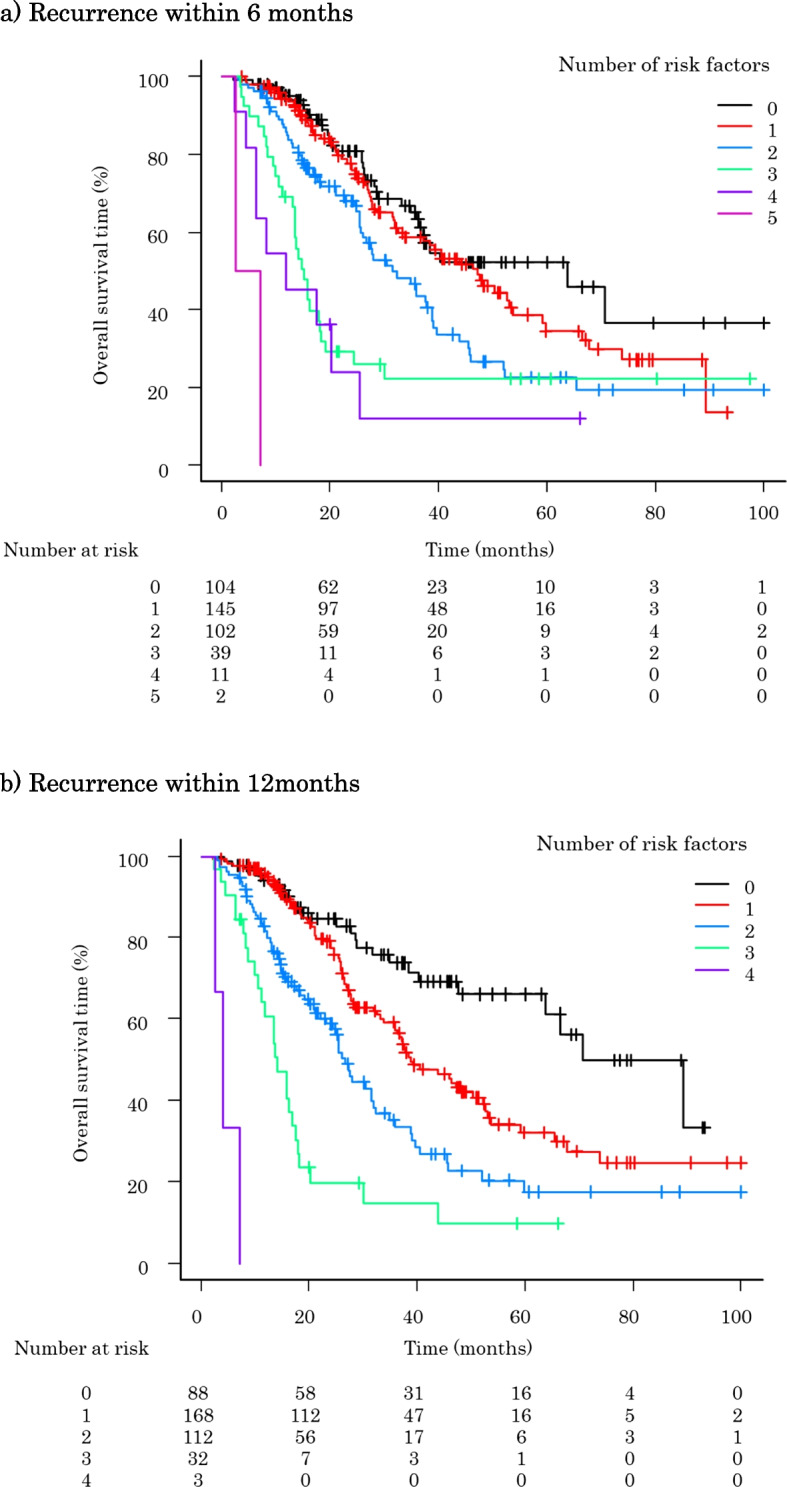
Relationship between the number of risk factors for recurrence within 6 and 12 months and the survival rate after surgery. **a** Recurrence within 6 months. **b** Recurrence within 12 months

The OS curves after pancreatic resection are shown according to the number of risk factors for recurrence within 6 or 12 months (Fig. 3). Patients with a higher number of risk factors had a significantly poorer OS in both the 6- and 12-month recurrence groups than those with fewer risk factors. When a patient had all risk factors, the median OS after surgery was 4.9 months for patients who experienced recurrence within 6 months and 4.0 months for those who experienced recurrence within 12 months.

## Discussion

In the current study, we found that 14.6% of PDAC patients who underwent curative-intent resection with or without preoperative multimodal therapy experienced recurrence within 6 months, and 37.9% experienced recurrence within 12 months. Earlier recurrence was associated with a poorer OS after surgery. We identified several independent risk factors for early recurrence; specifically, the preoperative CA19-9 level, tumor size, postoperative complications, positive peritoneal cytology, and the absence of adjuvant chemotherapy were associated with recurrence within 6 months, while the preoperative CA19-9 level, positive peritoneal cytology, lymph node metastasis, and the absence of adjuvant chemotherapy were risk factors for recurrence within 12 months. In addition, we found that a preoperative CA19-9 level > 175.8 U/mL, positive peritoneal cytology, and the absence of adjuvant chemotherapy consistently predicted early recurrence within either 6 or 12 months.

The development of early recurrence was associated with a shorter OS after surgery and a shorter post-recurrence survival [16–18]. Our study also showed that an earlier recurrence after surgery was associated with a poorer OS. Preoperative adjuvant therapy is a treatment option considered before surgery to help reduce the risk of micrometastases and ensure negative surgical margins. It can also provide important information about the tumor’s biology and help determine which patients may not be suitable for surgery. It may play an important role in overcoming early recurrence [12, 13]. In patients with early recurrence, the rate of liver metastasis (45.8%) was higher in the group with recurrence within 6 months than in the other groups, while the frequency of local and lymph node metastasis was higher in the late recurrence groups. This finding is consistent with previous reports of liver metastasis affecting 47.2 to 51.7% of cases with early recurrence within 6 months [16, 17]. Recurrence patterns are also related to the prognosis, as patients with metastatic disease localized to the lung have been shown to have a significantly longer survival than those with metastatic disease localized to the liver [15].

Serum CA19-9 is a crucial tumor marker for PDAC, and several reports have validated its clinical utility in the diagnosis and monitoring of disease progression [23], as well as in predicting early recurrence after surgery for PDAC. Previous studies identified preoperative CA19-9 as an important predictor for early recurrence either within 6 or 12 months for patient cohorts who underwent upfront surgery as well as those who received neoadjuvant chemoradiotherapy [16, 17, 19]. In line with previous studies, our study also showed that preoperative CA19-9 levels were able to predict early recurrence within 6 and 12 months, including in patients who received preoperative therapies. The preoperative CA 19–9 threshold for predicting early recurrence was 175.8 U/mL, which is similar to the range of 120 to 529 U/mL reported for predicting early recurrence [23]. However, it should be noted that the serum CA19-9 level may not be useful in patients who are Lewis antigen negative, which occurs at a rate of 5 to 10%.

We found that positive peritoneal cytology was an independent risk factor for early recurrence within 6 or 12 months in both all patients and those who received preoperative treatment. Given the poor prognosis for patients with cytological peritoneal metastasis after pancreatic resection, positive peritoneal cytology is considered a form of distant metastasis in the NCCN guidelines [10]. Ariake et al. reported that neoadjuvant therapy followed by surgery improved the OS in patients with cytological peritoneal metastasis compared to those who underwent upfront surgery [24]. This finding suggests that NAC may be effective in treating cytological peritoneal metastases and improving patient outcomes. Although our study did not detect any statistically significant differences, patients who received neoadjuvant treatment had a lower rate of early recurrence than those without such treatment. Therefore, a further investigation is needed to determine the efficacy of NAC for cytological peritoneal metastases.

Interestingly, our study revealed that the OS after surgical resection for potentially resectable PDAC gradually worsened according to the number of risk factors in the patient. We detected 5 independent risk factors for recurrence within 6 months and 4 risk factors for recurrence within 12 months. As the number of risk factors increased, the OS worsened, and for patients who had all of the risk factors, the OS after surgery was dismal. Calculating the number of risk factors would provide helpful information for predicting patients’ clinical outcomes and revising oncological management from initially planned adjuvant treatment strategies.

Several limitations associated with the present study warrant mention. First, this study had a retrospective design and single-center setting. Second, there is some bias, as the study does not take into consideration the changes in the treatment of PDAC over time, particularly in the regimens of adjuvant chemotherapy and selection of patients for neoadjuvant therapies.

In conclusion, the patients who suffered early recurrence either within 6 or 12 months after surgery were found to have worse prognoses than those patients either with recurrence after 12 months or with no recurrence at all. We clarified several factors associated with early recurrence, and the preoperative CA19-9 level, the presence of cytological peritoneal metastasis, and the lack of adjuvant chemotherapy were consistent predictors for early recurrence within 6 and 12 months. In addition, the number of risk factors affecting the patient was positively correlated with the OS after potentially curable resection. Calculating the number of risk factors for early recurrence in patients after surgery might be useful for predicting outcomes and help manage patients with a high risk for poor outcomes.

## Data Availability

Data will be made available on request.
